# Complete Genome Sequence of Bacillus velezensis LABIM40, an Effective Antagonist of Fungal Plant Pathogens

**DOI:** 10.1128/genomeA.00595-18

**Published:** 2018-06-21

**Authors:** Julia Pezarini Baptista, Paula Pinheiro Sanches, Gustavo Manoel Teixeira, Alexandre Tadachi Morey, Eliandro Reis Tavares, Sueli Fumie Yamada-Ogatta, Sérgio Paulo Dejato da Rocha, Mariangela Hungria, Renan Augusto Ribeiro, Maria Isabel Balbi-Peña, Roberta Torres Chideroli, Ulisses de Padua Pereira, Admilton Gonçalves de Oliveira

**Affiliations:** a Department of Microbiology, Universidade Estadual de Londrina (UEL), Londrina, Paraná, Brazil; b Embrapa Soja, Soil Biotechnology Laboratory, Londrina, Paraná, Brazil; c Department of Agronomy, UEL, Londrina, Paraná, Brazil; d Department of Preventive Veterinary Medicine, UEL, Londrina, Paraná, Brazil; e Laboratory of Electron Microscopy and Microanalysis, UEL, Londrina, Paraná, Brazil

## Abstract

Bacillus velezensis strain LABIM40 holds high potential for biological control of plant pathogens. Its complete genome contains one chromosome of 3,972,310 bp with 3,777 DNA coding sequences and displays 33 gene clusters potentially involved in the suppression of fungal pathogens.

## GENOME ANNOUNCEMENT

The increasing demand for safe and sustainable food supplies requires an efficient control of the major plant diseases. Current management practices are largely based on the application of synthetic pesticides, but their massive use has caused serious environmental impact and human and animal health concerns. Therefore, there is need for new environmentally friendly technologies and products to replace partly or fully chemical-based pesticides, contributing to safer crop disease control (1).

The use of plant pathogen antagonists for biological control has increased significantly, particularly in the last decade. Aerobic endospore-forming bacteria, such as Bacillus spp., are present in soil microbial communities and are known to produce structurally diverse metabolites with various biological effects, including antimicrobial activity against plant pathogens and other plant growth-promoting properties (2). The metabolites produced by Bacillus spp. include aminoglycosides, polyketides, and several small ribosomally and nonribosomally synthesized peptides, such as bacteriocins, oligopeptides, and lipopeptides, all of which have important roles in suppressing fungal diseases (3–5).

In this study, we report the complete genome sequence of Bacillus velezensis LABIM40, isolated as an antagonist contaminant of Fusarium oxysporum *in vitro* in the city of Londrina, Brazil; the strain was deposited at the Microbial Collection of the Microbial Biotechnology Laboratory, Universidade Estadual de Londrina. The cell-free supernatant of the bacterium strongly inhibits the growth of the important fungal plant pathogens Sclerotinia sclerotiorum, Rhizoctonia solani, Botrytis cinereal, and Macrophomina phaseolina (6), confirming its high biotechnological potential for biological control.

The Gentra Puregene genomic DNA kit (Qiagen, Brazil) was used for genomic DNA extraction. The B. velezensis LABIM40 genome was sequenced on the MiSeq platform, using a MiSeq version 3 reagent kit (600-cycle, Illumina, Brazil) at Embrapa Soja, Londrina. After we sequenced them, the reads were subjected to trimming and filtering using CLC Genomics Workbench version 10.0. Reads with an average Phred quality value of less than 30 and with one or more ambiguities were removed; reads smaller than 50 bp, as well as the last 10 nucleotides of the 3′ end of each read, were also removed. Sequencing allowed a genome coverage of 212-fold, assembled into 19 contigs with CONTIGuator software (7). The genome was compared with other genomes of the same species, and the gaps were removed with recursive rounds of short reads mapped against the scaffold (8). The annotation was created using the NCBI Prokaryotic Genome Annotation Pipeline (PGAP). The genome was estimated at 3,972,310 bp, with a G+C content of 46.5%, and the average nucleotide identity with its closest strain, B. velezensis S141 (9), was 99.1%. The genome of LABIM40 harbors 3,777 DNA coding sequences, 7 rRNA operons, 75 tRNAs, and 105 pseudogenes. Thirty-three putative gene clusters responsible for secondary metabolite biosynthesis were identified by using antiSMASH version 4.1.0 (10). Among them, we can highlight thiopeptides, polyketide synthase antibiotics, nonribosomal peptide synthetase antibiotics, bacteriocins, and terpenes, which may be responsible for the antifungal activity. The genome of B. velezensis LABIM40 may help to explore the metabolic pathways related to its antimicrobial activity, emphasizing its biotechnological potential.

### Accession number(s).

This whole-genome project was deposited at the DDBJ/EMBL/GenBank under the accession number CP023748 (BioProject PRJNA412668, BioSample SAMN07722662). The version described in this paper is the first version, CP023748.1.
